# Steroid metabolites for diagnosing and predicting clinicopathological features in cortisol-producing adrenocortical carcinoma

**DOI:** 10.1186/s12902-020-00652-y

**Published:** 2020-11-23

**Authors:** Sawako Suzuki, Tomoki Minamidate, Akina Shiga, Yutarou Ruike, Kazuki Ishiwata, Kumiko Naito, Akiko Ishida, Hanna Deguchi, Masanori Fujimoto, Hisashi Koide, Ichiro Tatsuno, Jun-ichiro Ikeda, Yuto Yamazaki, Hironobu Sasano, Koutaro Yokote

**Affiliations:** 1 Department of Endocrinology, Hematology and Gerontology, Chiba University Graduate School of Medicine, 1-8-1 Inohana, Chuo-ku, Chiba, 260-8670 Japan; 2 Department of Diabetes, Metabolism and Endocrinology, Chiba University hospital, Chiba, Japan; 3 Center for Diabetes, Metabolism and Endocrinology, Toho University Sakura Medical Center, Sakura, Japan; 4 Department of Diagnostic Pathology, Chiba University Graduate School of Medicine, Chiba, Japan; 5 Department of Pathology, Tohoku University School of Medicine, Sendai, Japan

**Keywords:** Adrenocortical carcinoma, Steroid metabolites, Cushing’s syndrome, Clinicopathology

## Abstract

**Background:**

Approximately 60% of adrenocortical carcinomas (ACC) are functional, and Cushing’s syndrome is the most frequent diagnosis that has been revealed to have a particularly poor prognosis. Since 30% of ACC present steroid hormone-producing disorganization, measurement of steroid metabolites in suspected ACC is recommended. Previous reports demonstrated that steroid hormone precursors or their urine metabolites, which can be assessed using liquid chromatography tandem mass spectrometry (LC-MS/MS) or gas chromatography mass spectrometry (GC-MS) respectively, are useful for distinguishing ACC from cortisol-producing adenomas (CPA); however, despite high precision, LC-MS/MS and GC-MS require a highly trained team, are expensive and have limited capacity.

**Methods:**

Here, we examined 12 serum steroid metabolites using an immunoassay, which is a more rapid and less costly method than LC-MS/MS, in cortisol-producing ACC and CPA. Further, the correlation of each steroid metabolite to the classification stage and pathological status in ACC was analyzed.

**Results:**

Reflecting disorganized steroidogenesis, the immunoassay revealed that all basal levels of steroid precursors were significantly increased in cortisol-producing ACC compared to CPA; in particular, 17-hydroxypregnenolone (glucocorticoid and androgen precursor) and 11-deoxycorticosterone (mineralocorticoid precursor) showed a large area under the ROC curve with high sensitivity and specificity when setting the cut-off at 1.78 ng/ml and 0.4 mg/ml, respectively. Additionally, a combination of androstenedione and DHEAS also showed high specificity with high accuracy. In cortisol-producing ACC, 11-deoxycortisol (glucocorticoid precursor) showed significant positive correlations with predictive prognostic factors used in ENSAT classification, while testosterone showed significant positive correlations to the Ki67-index in both men and women.

**Conclusion:**

Less expensive and more widely available RIA and ECLIA may also biochemically distinguish ACC from CPA and may predict the clinicopathological features of ACC.

## Background

Adrenocortical carcinomas (ACC) are rare but aggressive endocrine neoplasms from the adrenal cortex. ACC may be functional and cause Cushing’s syndrome and/or virilization [1–3], and it may present as an abdominal mass or incidental findings. Further, approximately 30% of ACC are responsible for multiple hormone production, including steroid hormone precursors, which is referred to as disorganized steroidogenesis [4]. The ACC prognosis is poor, and the 5-year overall survival is lower than 35% in most studies [5, 6]. Management is multidisciplinary and includes surgical resection, oral mitotane, intravenous chemotherapy, and palliative radiation [7]. Therefore, early diagnosis, accurate staging, and appropriate treatment according to progression prediction are important. For accurate determination, the combination of tumor size (≥65 mm) [8] and careful pathological investigation with the Weiss score (≥3 out of 9) [8] and Ki67-index (≥10%: high risk) [9] is widely used for ACC diagnosis. The European Network for the Study of Adrenal Tumors (ENSAT) system is used for ACC staging classification [9]. Both the growth marker Ki67 and ENSAT classification are also used as predicting prognostic factors of ACC [9]; however, image-guided adrenal biopsy or adrenalectomy necessary for pathological diagnosis is invasive and cannot be performed in patients with a poor general condition. Additionally, adrenal biopsy is an expensive procedure with a reported rate of nondiagnostic biopsies of 8.7%, a complication rate of 2.5% [10], and lower accuracy with 70% sensitivity and 98% specificity [11]. Therefore, less invasive and less costly methods for the diagnosis and prognostic prediction of ACC, along with repeat imaging, are desired for the determination of appropriate treatment. Recently, steroid profiling has emerged as a powerful novel diagnostic tool for ACC [11–14]. Androgen-producing ACC is easy to diagnose because cortisol-producing adenomas (CPA) very rarely produce androgens. Conversely, in non-androgen producing ACC, the differentiation between ACC and CPA becomes challenging because the end-product cortisol presents similarly between ACC and CPA. Previous reports demonstrated that serum steroid metabolite examination using liquid chromatography tandem mass spectrometry (LC-MS/MS) [12, 13] or urine steroid metabolites using gas chromatography mass spectrometry (GC-MS) [15–21] are useful for distinguishing ACC from adrenocortical adenoma. Furthermore, the ENSAT recommends a biochemical workup for suspected ACC that includes serum cortisol, aldosterone, 17-hydroxyprogesterone, dehydroepiandrosterone sulfate (DHEAS), androstenedione, testosterone, and 17-beta-estradiol (http://www.ensat.org/page-1317312); however, feasible methods to detect or predict ACC behavior and prognosis aside from LC-MS/MS or GC-MS are limited by their lack of clinical availability. In the present study, we aimed to compare the steroidal metabolic profile in cortisol-producing ACC and CPA and to correlate steroid metabolites with clinical, pathological and prognostic parameters using less expensive and more widely available assays (RIA and ECLIA).

## Methods

### Study population

This study was approved by the Human Research Ethics Committee at Chiba University (approval number: 828 and 3957), and all patients provided written informed consent. We prospectively analyzed the steroid profiles of 7 patients with cortisol-producing ACC and 25 CPA patients who were admitted to our hospital between 2013 and 2018. The final diagnosis had been ascertained by histology and evidence of metastasis in ACC, and by imaging, biochemical, and clinical follow-up showing no evidence of adrenal tumor growth and metastasis in CPA. Endocrinologically, overt Cushing’s syndrome was determined in patients with signs or symptoms of excess hormones, increased 24-h urinary free cortisol, and high plasma cortisol that could not be suppressed below 5 μg/dl with overnight dexamethasone at doses of 1 mg. Subclinical Cushing’s syndrome was determined in patients with no signs or symptoms of excess hormones, subnormal suppression following overnight dexamethasone (1 mg; > 1.8 μg/dl), and normal 24-h urinary free cortisol [22, 23].

### Clinical evaluation

We analyzed five important clinicopathological parameters in cortisol-producing ACC: ENSAT classification, Weiss score, Ki67-index derived by pathological immunohistological staining, tumor size, and overall survival. We further examined the correlation of these five clinical parameters to steroid metabolites.

Then, we identified the steroid metabolites that correlated with pathological findings or clinical parameters related to staging and disease prognosis in ACC. The ENSAT staging system consists of stages I (T1N0M0), II (T2N0M0), III (T1–2N1M0 or T3–4N0–1M0), and IV (TanyNanyM1, metastatic ACC) [24]. The Weiss score is comprised of nine histological criteria: (i) high nuclear grade, (ii) mitotic rate greater than five per 50 high power fields (HPF), (iii) atypical mitotic figures, (iv) eosinophilic tumor cell cytoplasm (greater than 75% of tumor cells), (v) diffuse architecture (greater than 33% of the tumor), (vi) necrosis, (vii) venous invasion, (viii) sinusoidal invasion, and (ix) capsular invasion [25]. A tumor is labeled malignant when it meets three or more of these histological criteria [8]. The Ki67-index is evaluated using an immunohistochemical assessment of cell proliferation by the detection of Ki67 antigen in neoplastic cell populations; a Ki67-index of 10% or more is diagnosed as high risk [9].

### Clinical samples and hormonal assays

We compared 12 serum steroid metabolites using an immunoassay, which is a more rapid and less expensive method compared to LC-MS/MS, in cortisol-producing ACC and CPA. In addition, 24-h urine excretion of individual 17-ketosteroids as androgen secretions were examined by GC-MS for comparative analysis. These serum and urine metabolites, with the exception of the largest produced end products (cortisol, aldosterone, and DHEAS), were not regularly measured but examined in all subjects in our study before starting treatment with mitotane, ketoconazole, metopirone, or other drugs that influence the hormonal evaluation. Under basal conditions, fasting blood was withdrawn after a 15 min rest between 8:00 and 9:00 AM. The day before, 24-h urine specimens were collected for periods of two to 3 days, and urinary free cortisol and 17-ketosteroid fractions were measured. Urinary and serum cortisol were measured by radioimmunoassay (RIA). 11-deoxycortisol, 11-deoxycorticosterone, corticosterone, aldosterone, pregnenolone, 17-hydroxypregnenolone, and androstenedione were measured by RIA. Progesterone, 17-hydroxyprogesterone, and testosterone were measured by electrochemical luminescence immunoassay (ECLIA). Urinary 17-ketosteroid fractions were measured by GC-MS. Sample analysis was completed by a Japanese clinical analytical laboratory (SRL, Inc., Tokyo, Japan).

### Statistical analyses

Shapiro-Wilk test showed that the endocrinological data were not normally distributed. Hence, pairwise comparisons were performed using the Mann-Whitney U-test. The results were expressed as a median (interquartile ranges), and a value of *P* < 0.05 was considered statistically significant. The Chi-Square statistic is used for testing relationships between categorical variables. Receiver operating characteristics (ROC) curves were generated for steroid metabolites that displayed relatively significant differences between ACC and CPA. The area under the ROC curve (AUC) was also calculated. A perfect classifier has AUC = 1, and a completely random classifier has AUC = 0.5. Sensitivity and specificity were calculated at cut-off values providing the highest sensitivity. The correlation between the clinicopathological parameters and individual steroid metabolites in ACC was calculated by Pearson’s correlation coefficient (R). R values between 0.7 and 1.0 together with *P* < 0.05 can be considered highly correlated. Statistical analysis was performed using SPSS Statistics for Windows (SPSS Inc., Chicago, IL, USA).

## Results

### Patients and clinical characteristics

We evaluated 7 cortisol-producing ACC patients and 25 CPA patients as shown in Table 1. The ACC population was older than the CPA population, while gender and cortisol producing ability evaluated by urinary cortisol and the dexamethasone suppression test did not differ between two groups. Four of 7 ACC patients and 11 of 25 CPA patients presented with signs of Cushing’s syndrome and were diagnosed as having overt Cushing’s syndrome. Three of 7 ACC patients and 2 of 25 CPA patients showed high testosterone levels relative to age- and sex-matched controls. All ACC patients and 80% of CPA patients underwent adrenalectomy and were pathologically diagnosed. Adrenal tumor size and Weiss scores were higher in the ACC than CPA patients. The median Ki67 index was 23 in ACC. Of the ACC patients, three were in stage II, one in stage III, and three in stage IV at diagnosis. The overall survival of ACC patients varied from 5 to 60 months.

**Table 1:** Endocrinological characteristics of ACC and CPA

Variable	Unit	ACC (*n* = 7)		CPA (*n* = 25)		*P*-value
		Overt Cushing’s syndrome	Subclinical Cushing’s syndrome	Overt Cushing’s syndrome	Subclinical Cushing’s syndrome	
Number of patients	n (%)	4 (57%)	3 (43%)	11 (44%)	14 (56%)	NS
Basal ACTH levels	pg/ml	2.6 (0–5.97)	5.5 (2.75–11.6)	0 (0–0)	10.05 (1.67–13.67)	
Basal cortisol levels	μg/ml	22.45 (12.7–33.4)	8 (5.9–10.65)	17.6 (15.6–18.35)	11.15 (9.8–13.87)	
High testosterone levels relative to normal ranges	n (%)	3 (43%)		2 (8%)		< 0.05
Basal testosterone levels	ng/ml	Male: 2.29 (1.27–3.30) Female: 1.3 (0.36–1.97)		Male: 5.61 (4.42–6.64) Female: 0.19 (0.15–0.26)		
Age	years	67 (66–71.5)		56 (45–63)		< 0.05
Gender	Male/ Female	2 / 5		4 / 21		NS
Post-overnight 1 mg dexamethasone suppression serum cortisol	*μg/dl*	16.4 (10.65–21.15)		10.3 (3.87–15.8)		NS
Post-overnight 8 mg dexamethasone suppression serum cortisol	μg/dl	16.2 (10.6–21.55)		12.05 (4.75–18.03)		NS
Urinary free cortisol	μg/day	128.15 (59.63–148)		71.5 (29.7–267)		NS
Tumor size	mm	80.5 (61.25–82.5)		28 (22–33)		< 0.01
Surgical removal of adrenal tumor	n (%)	7 (100%)		20 (80%)		NS
Weiss score		5.5 (5–6.75)		0 (0–1)		< 0.001
Ki67-index	%	23 (20.75–28.25)				
ENSAT	stage	3 (2–4)				
Overall survival	months	25 (17–50)				

Data were expressed as median (interquartile ranges). *P* < 0.05 was defined as significant. *NS* nonsignificant, *ACC* adrenocortical carcinomas, *CPA* cortisol-producing adenomas

### Serum concentration and urinary excretion of steroid metabolites

**Fig. 1:**
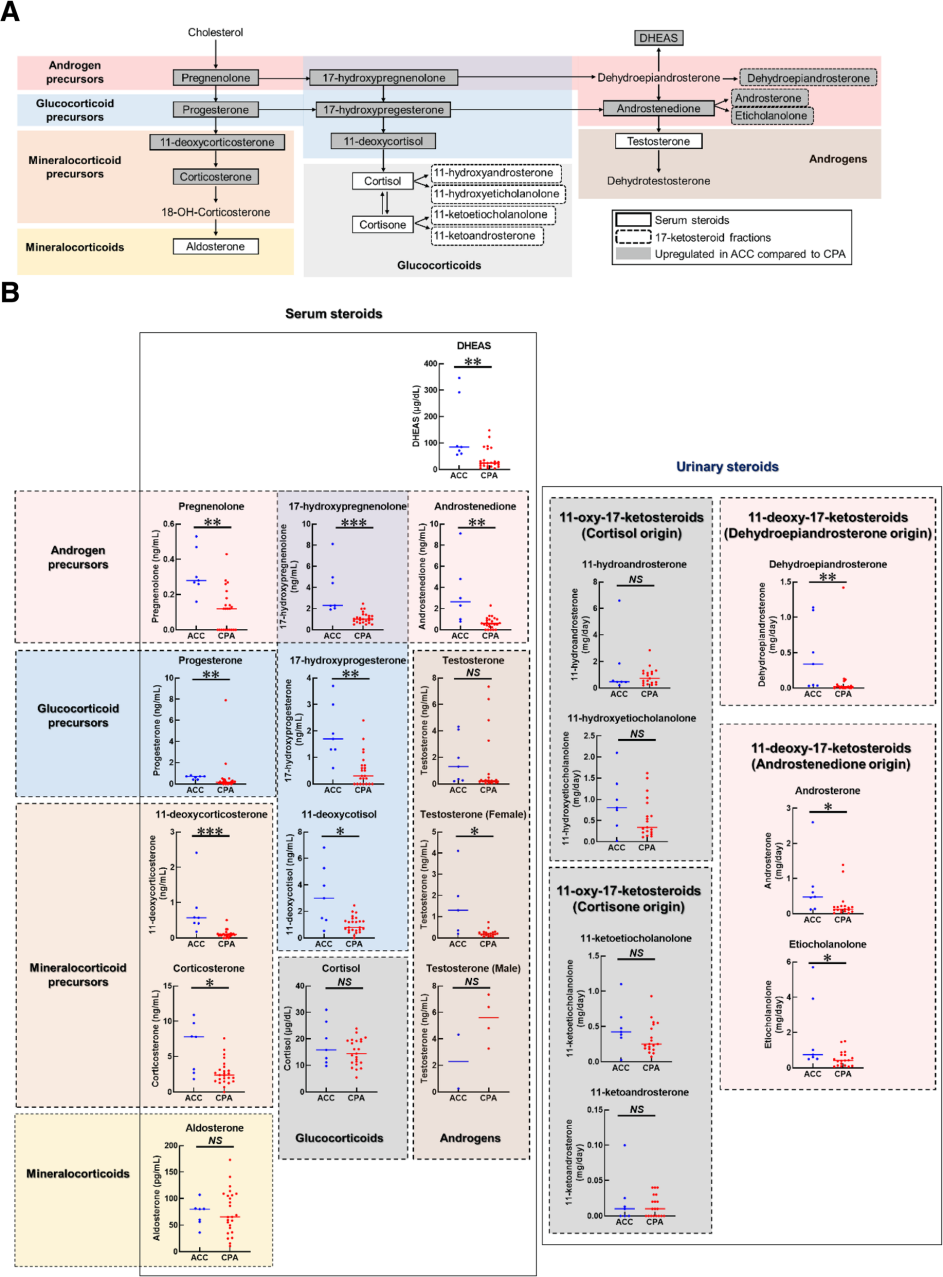
**a** Steroid pathway diagram indicating the observed changes in steroid metabolite concentrations in ACC patients compared to CPA patients. DHEAS: dehydroepiandrosterone sulfate. **b** Comparison of indicated serum concentrations of steroids and urinary 17-ketosteroid excretion between ACC and CPA. DHEAS: dehydroepiandrosterone sulfate. ACC: adrenocortical carcinomas, CPA: cortisol-producing adenomas. *P* < 0.05 was defined as significant. *: < 0.05, **: < 0.01, *** < 0.001, NS: nonsignificant

**Fig. 2:**
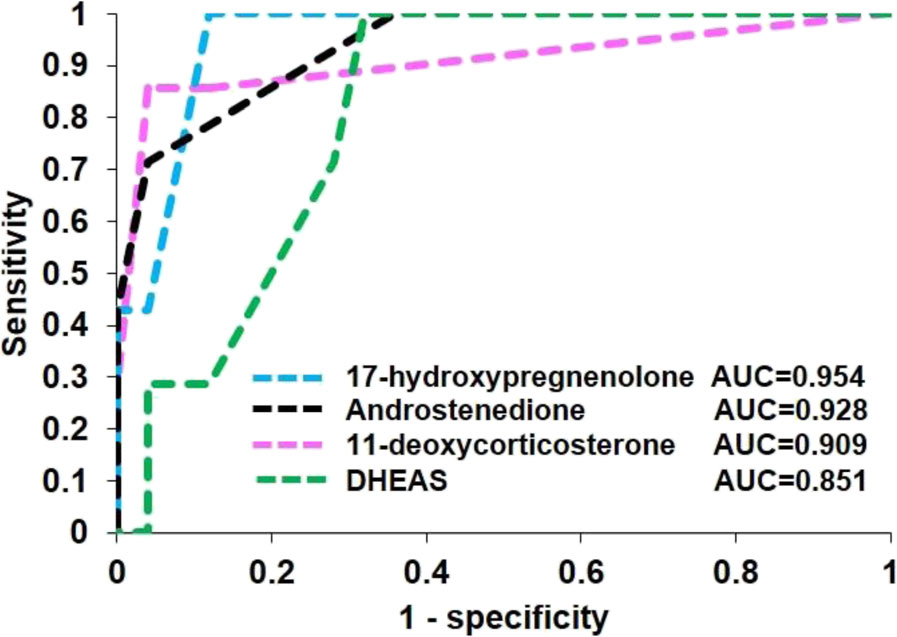
Receiver operating characteristic (ROC) curve for 11-deoxycorticosterone, 17-hydroxypregnenolone, androstenedione, and dehydroepiandrosterone sulfate (DHEAS) for the diagnosis of ACC

Comparison of serum steroid metabolites and urinary metabolites between cortisol-producing ACC and CPA is shown in Fig. 1. The comparison of the profiles and outcomes of basal serum and urinary 17-ketosteroids between ACC and CPA patients are summarized in the steroid pathway diagram (Fig. 1a). Comparison of the basal steroid profiles of ACC and CPA patients revealed striking differences. Significantly higher levels of glucocorticoid precursors (progesterone, 17-hydroxyprogesterone, and 11-deoxycortisol), mineralocorticoid precursors (11-deoxycorticosterone and corticosterone), androgen precursors (pregnenolone, 17-hydroxypregnenolone, and androstenedione), and DHEAS were observed in ACC patients compared to CPA patients (Fig. 1b). No significant difference was found in glucocorticoid, mineralocorticoid, and androgen itself (Fig. 1b). The urine 17-ketosteroid fractions of 11-deoxy-17-ketosteroid (androsterone, etiocholanolone, and dehydroepiandrosterone) derived from androgen precursors including androstenedione and DHEA showed a significant increase in ACC patients compared to CPA patients (Fig. 1b). These data demonstrated that ACC patients exhibited steroid disorganization as previously reported, and immunoassay is useful enough for distinguishing ACC from CPA. Next, steroid metabolites were plotted on an ROC curve, and then the area under the ROC (AUC) as well as the most appropriate cut-off values were calculated to classify cortisol-producing ACC or CPA. ROC-analysis demonstrated that 12 steroid metabolites had a sensitivity > 85% for detecting ACC (Table 2). The ROC curves for 17-hydroxypregnenolone, androstenedione, and 11-deoxycorticosterone were found to have a large AUC (0.954, 0.928, and 0.909, respectively) (Table 2 and Fig. 2). Setting the cut-off at 1.78 ng/ml for 17-hydroxypregnenolone revealed 100% sensitivity and 88% specificity (Table 2). 11-deoxycorticosterone showed 86% sensitivity and 96% specificity at a cut-off value of 0.4 mg/ml (Table 2). Although each androstenedione or DHEAS had low specificity and low accuracy (Table 2), the combination of DHEAS and androstenedione using each cut-off value (32.5 μg/dl and 1.52 ng/ml, respectively) showed high specificity (67% sensitivity and 100% specificity) with high accuracy (0.935). Based on these results, 17-hydroxypregnenolone, 11-deoxycorticosterone as well as the combination of DHEAS and androstenedione were considered the strongest indicators for the detection of cortisol-producing ACC among the analyzed serum steroid metabolites.

**Table 2:** Receiver operating characteristics (ROC) for steroid metabolites with sensitivity for detecting ACC > 85%

	AUC	Cuff-off value	Unit	Sensitivity (%)	Specificity (%)	Accuracy
Serum steroids						
17-hydroxyprogesterone	0.898	0.62	ng/mL	100	52	0.633
11-deoxycortisol	0.789	1.14	ng/mL	86	52	0.594
Cortisol	0.602	9.75	μg/dL	100	17	0.367
11-deoxycorticosterone	0.909	0.40	ng/mL	86	96	0.938
Corticosterone	0.800	1.82	ng/mL	100	28	0.438
Aldosterone	0.494	24.04	pg/mL	100	8	0.290
Pregnenolone	0.898	0.133	ng/mL	100	65	0.733
17-hydroxypregnenolone	0.954	1.78	ng/mL	100	88	0.906
Androstenedione	0.928	1.52	ng/mL	100	64	0.710
DHEAS	0.851	32.50	μg/dL	100	71	0.774
Urinary steroids						
11-hydroxyetiocholanolone	0.489	0.37	mg/day	86	53	0.615
11-ketoetiocholanolone	0.514	0.29	mg/day	86	58	0.654

*ACC* adrenocortical carcinomas, *DHEA-S* dehydroepiandrosterone sulfate, *AUC* area under the curve

### Clinical correlation to steroid metabolites in ACC

**Fig. 3:**
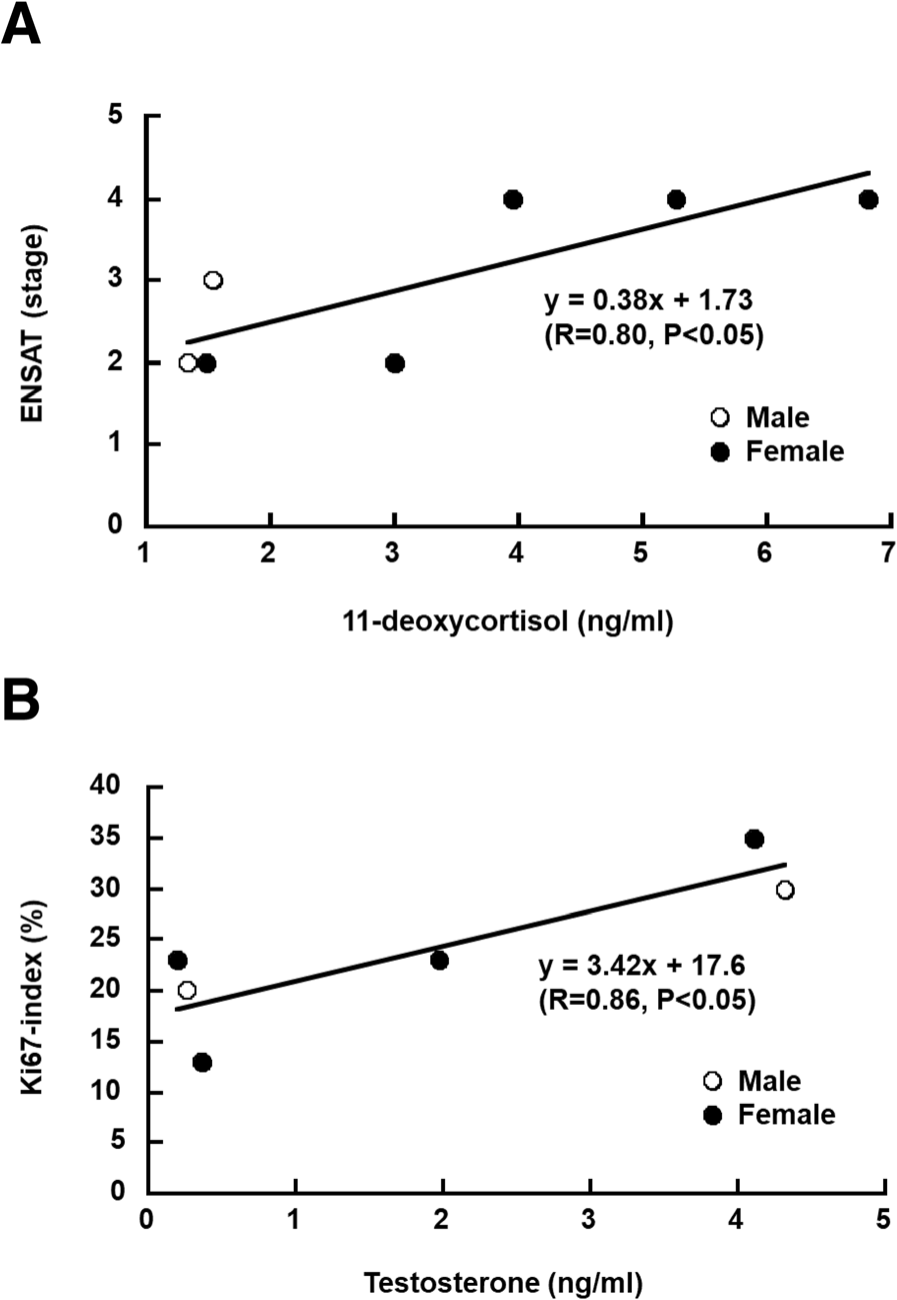
**a** and **b** Scatter plot highlighting the correlation between basal 11-deoxycortisol and ENSAT (**a**) and testosterone and Ki67-index (**b**) in ACC patients. White circles and black circles indicate male and female, respectively

To investigate whether serum steroid metabolites can be used as prognostic factors in cortisol-producing ACC, we analyzed the correlation between serum steroid metabolites and clinical parameters including tumor size, ENSAT classification, Weiss score, Ki67-index, and overall survival in ACC (Table 3). The correlation between urinary 17-ketosteroids and clinical parameters were also examined as a comparative analysis (Table 3). The serum levels of 11-deoxycortisol showed a significant positive correlation with the ENSAT classification (*R*-value: 0.80, *P*-value: 0.03) (Table 3 and Fig. 3a). Further, the testosterone serum levels showed a significant positive correlation with the Ki67-index (*R*-value: 0.86, *P*-value: 0.03) (Table 3 and Fig. 3b). Although a gender difference in testosterone was found, a correlation between testosterone and Ki67 was observed in both men and women, and was considered to be on the same correlation line (Fig. 3b). No significant correlation was found between the Weiss score, tumor size, or overall survival, and any steroid metabolites. Further, no significant correlation was found between urinary 17-ketosteroids and any of the clinical parameters.

**Table 3:** The correlation coefficient (R) and statistical significance (*p*-value) for steroids and clinicopathological parameters in ACC

	ENSAT		Weiss score		Ki-67 index		Tumor size		Overall survival	
	*R*-value	P-value	*R*-value	*P*-value	*R*-value	*P*-value	*R*-value	*P*-value	*R*-value	*P*-value
Serum steroids										
Progesterone	−0.11	0.82	−0.12	0.82	0.09	0.86	−0.41	0.42	0.21	0.66
17-hydroxyprogesterone	0.40	0.37	0.49	0.33	0.58	0.23	−0.46	0.36	− 0.57	0.19
11-deoxycortisol	**0.80**	**0.03**	− 0.71	0.12	0.17	0.75	0.38	0.46	0.28	0.54
Cortisol	−0.14	0.79	0.53	0.27	−0.48	0.28	0.19	0.71	0.57	0.32
11-deoxycorticosterone	0.54	0.21	−0.79	0.06	0.02	0.97	0.62	0.19	0.42	0.34
Corticosterone	−0.01	0.98	−0.70	0.12	0.00	1.00	0.31	0.55	−0.06	0.90
Aldosterone	0.55	0.20	−0.11	0.84	0.79	0.06	−0.18	0.73	−0.30	0.51
Pregnenolone	−0.03	0.96	0.08	0.89	0.60	0.21	0.25	0.64	0.04	0.94
17-hydroxypregnenolone	−0.01	0.99	0.77	0.07	0.33	0.53	−0.41	0.42	−0.29	0.53
Androstenedione	0.33	0.52	0.12	0.85	0.03	0.96	0.66	0.23	0.20	0.70
Testosterone	0.28	0.54	0.44	0.38	**0.86**	**0.03**	−0.21	0.69	−0.65	0.11
DHEAS	−0.10	0.83	0.58	0.23	0.07	0.89	0.16	0.77	0.29	0.52
Urinary steroids										
11-hydroandrosterone	0.33	0.47	0.52	0.29	−0.19	0.72	0.16	0.76	−0.05	0.92
11-hydroxyetiocholanolone	−0.15	0.75	0.60	0.21	−0.56	0.25	0.26	0.62	0.12	0.79
11-ketoandrosterone	0.33	0.46	0.13	0.80	−0.46	0.36	0.09	0.86	−0.03	0.94
11-ketoetiocholanolone	−0.01	0.98	0.42	0.41	−0.68	0.14	0.13	0.80	0.26	0.57
Androsterone	0.49	0.26	0.45	0.37	0.56	0.24	0.12	0.82	−0.15	0.75
Etiocholanolone	0.15	0.74	0.60	0.21	−0.02	0.98	0.19	0.71	0.15	0.75
Dehydroepiandrosterone	0.14	0.76	0.60	0.21	0.35	0.50	0.21	0.69	0.02	0.96

*DHEA-S* dehydroepiandrosterone sulfate, *R*-values with *P* < 0.05 printed in boldface

## Discussion

In the present study, all basal levels of steroid precursors and DHEAS were significantly increased in cortisol-producing ACC patients compared to CPA patients. The immunoassay was a successful tool for diagnosis, and we recommend 17-hydroxypregnenolone (glucocorticoid and androgen precursor), 11-deoxycorticosterone (mineralocorticoid precursor), or the combination of DHEAS and androstenedione (androgen precursor) for the diagnosis of cortisol-producing ACC. Notably, the combination of DHEAS and androstenedione could be a valuable tool to differentiate ACC from CPA is of extreme relevance, since these are two widely available markers, and can be implemented without a major increase in costs. Only 17-ketosteroids were measured as urine steroid metabolites in our study, but none of the 17-ketosteroid fractions were sensitive to ACC diagnosis. Moreover, we propose that serum steroid metabolite measurement, especially 11-deoxycortisol (glucocorticoid precursor) and testosterone, as a simple and noninvasive method for predicting the progression and prognosis of patients with cortisol-producing ACC.

The observed heterogeneity of steroidogenesis reflects immature and dedifferentiated cell features, which are the hallmark of ACC [11, 13, 15–17]. To our knowledge, no serum or urinary steroid metabolites associated with prognostic factors in ACC have been demonstrated to date, but several papers have reported these were useful for the diagnosis of ACC [11–21]. Consistent with our recommendation, Schweitzer et al. reported that the combination of androgen precursor (17-hydroxypregnenolone, progesterone, and DHEA), mineralocorticoid precursor (11-deoxycorticosterone), glucocorticoid precursor (11-deoxycortisol), and sex hormone (DHEAS and estradiol) was useful for ACC diagnosis [13]. Taylor et al. also recommended androgen precursor (17-hydroxypregnenolone) and glucocorticoid precursor (11-deoxycortisol) for the diagnosis of ACC [12]. For urinary steroid metabolites, 11-deoxycortisol urinary metabolite tetrahydro-11-deoxycortisol (glucocorticoid precursor) [15–18] as well as 17-hydroxypregnenolone urine metabolite pregnanetriol and pregnenolone urine metabolite pregnanediol (androgen precursor) [17, 18] were recommended. Although all previous reports suggested that some steroid hormone precursors were useful in the diagnosis of ACC, the differences between the type of steroid precursors may be due to differences in the cortisol-producing ability within ACC and adrenocortical adenoma. Accurate 24-h collections are often not easy to obtain, and blood collections are more convenient for patients.

LC-MS/MS has less cross-reactivity to other metabolites with peptide specificity and high sensitivity, but it is expensive, requires more time for processing and measurement than immunoassay. On the other hand, immunoassay is less expensive and more wildly available, and thus can be performed in the largest number of places, including poorer countries that will unlikely have access to LC-MS/MS; however, immunoassay is not without concerns regarding the accuracy, level of validation, intra- and interassay and more details on the standardization of the measurement of each steroid using RIA and ECLIA, particularly for intermediate metabolites, which has few or almost no standardized methods other than LC-MS/MS or GC-MS. Therefore, the JCEM published an editorial in 2013 suggesting that the Journal policy should be to accept only articles that use mass spectrometry for sex steroid assays [26], which has been reinforced by a letter also published in JCEM [27]. These issues were discussed in detail at the 2014 Workshop on Measuring Estrogen Exposure and Metabolism. A workshop on measuring steroids, particularly estradiol, concluded that “both immunoassays and mass spectrometry-based assays for estrogens and their metabolites would be acceptable if they are accurate and reliable and meet performance criteria suitable for their intended use” [28]. Laurence M et al. mentioned that the debate will continue until mass spectrometry assays for steroid hormones become sufficiently rapid, inexpensive, and robust for general acceptance and use [29]. In this study, we revealed that RIA and ECLIA provide sufficient ability to distinguish ACC from CPA; however, lower concentrations lose accuracy and precision in RIA and ECLIA compared to LC-MS/MS, which may be a limitation. Because DHEAS is inexpensive, widely available for clinical use and is present in higher concentrations such that the absolute precision is comparable to LC-MS/MS, using androstenedione combined with DHEAS to distinguish ACC from CPA may overcome this limitation of RIA and ECLIA.

The reasons for the correlations between 11-deoxycortisol and ENSAT classification, or testosterone and KI67-index were not elucidated in our study, and we will therefore base our analysis on previous reports. Cortisol-producing ACC has been known to produce androgen the most simultaneously to cortisol [30–32]. Cortisol-producing ACC that produce male sex hormone at the same time might have higher cell proliferation. Because 11-deoxycortisol is converted to cortisol by Cytochrome P450 Family 11 Subfamily B Member 1 (CYP11B1), a relative deficiency of CYP11B1 may occur in ACC with poor progression. In fact, CYP11B1 expression has been shown to be downregulated in some ACC patients [33].

A confirmed diagnosis of ACC cannot be made is some cases, or surgery cannot be performed due to a poor general condition or severe complications. In such cases, the measurement of serum steroid hormone metabolites would be helpful for predicting pathological characteristics or prognosis in cortisol-producing ACC; however, our study is limited to cortisol-producing adrenal tumors without evaluation of estrogen-producing tumors is a prospective study with a small number of ACC samples and was limited to urinary hormone measurements. Additional large-scale prospective studies will be required in the future.

## Conclusion

Combined serum steroid metabolite measurement is a highly informative noninvasive method for diagnosing and predicting the clinicopathological features associated with the prognosis of cortisol-producing ACC.

## Data Availability

Data are available from the corresponding author upon reasonable request.
